# Effects of team-based mixed reality simulation program in emergency situations

**DOI:** 10.1371/journal.pone.0299832

**Published:** 2024-02-29

**Authors:** Moon-Ji Choi, Kyeng-Jin Kim

**Affiliations:** 1 Department of Nursing, Kyungil University, Gyeongsan, South Korea; 2 College of Nursing, Kyungpook National University, Daegu, South Korea; Ain Shams University Faculty of Nursing, EGYPT

## Abstract

**Background:**

This study aimed to demonstrate the effectiveness of a team-based mixed reality simulation program about emergencies.

**Method:**

A nonequivalent control group pretest-posttest design was utilized. We recruited 32 nurses for the experimental group and 32 for the control group, resulting in a total of 61 nurses ultimately included as subjects for analysis. This mixed reality program is designed to facilitate responses in cardiopulmonary resuscitation situations simultaneously using four HoloLens. With shared content visible to all four trainees, the participants could engage in simulation while freely communicating. The experimental group engaged in cardiopulmonary resuscitation emergency simulation while wearing the HoloLens, while the control group was provided with written CPR materials.

**Results:**

There were significant increases in the experimental group’s critical thinking (*p* < .001), learning transfer motivation (*p* = .006), communication confidence (*p* = .033), and learning immersion (*p* < .001) compared to the control group.

**Conclusion:**

The program developed in this study presents an effective educational strategy that can enhance nurses’ emergency competencies and leverage the practicality of mixed reality.

## Introduction

In medical environments, crises are encountered, and it is impossible to eliminate all risk factors. Continuous efforts and capacity enhancement are necessary to ensure an acceptable level of risk. Cardiac arrest in patients results from compromised heart functions, which result in a cessation of systemic blood circulation. This is an unpredictable and severe “crisis” that leads to death if more than 4 to 6 minutes elapse after its onset [1]. Despite recent advancements in medical technology, the incidence of sudden cardiac arrest is rapidly increasing. This crisis has emerged as a global health issue [2]. As primary healthcare providers offering care in proximity to patients, nurses often play a crucial role in responding to in-hospital cases of cardiac arrest [3]. Since cardiac arrest can occur at any time for a patient admitted to a hospital, nurses must be able to assess the patient’s situation quickly, perform basic cardiopulmonary resuscitation (CPR), and prepare advanced cardiac life support [4].

In various nursing scenarios, CPR varies depending on the department. However, as CPR is not a routine nursing activity, many nurses lack confidence and fear regarding such situations. According to research, nurses feel pressure, stress, and a lack of confidence in CPR interventions [5]. Additionally, performing CPR is hindered by a lack of knowledge, skills, and education, indicating barriers to its execution [6]. These difficulties contribute to nurses developing a negative attitude toward CPR, potentially leading to delays in its implementation [7].

CPR is a skill that even seasoned medical professionals find challenging to master consistently, despite their extensive years of experience. It is commonly recommended to undergo cardiopulmonary resuscitation (CPR) training and retraining every 1 or 2 years to maintain knowledge and skills [8]. Additionally, some hospitals have implemented guidelines to encourage annual CPR training for healthcare providers. However, it has been observed that knowledge and self-assurance in performing CPR decline significantly after six months of CPR education [9]. Recently, the need for immediate in-hospital CPR intervention underscores the importance of a team-based approach. Effective communication and collaborative skills among team members have emerged as essential prerequisites for successful CPR execution within the hospital setting.

Simulation education has garnered attention as an effective learning method for maintaining the efficacy of CPR training [10]. According to the theory proposed by Jeffries, Rodgers, and Adamson [11], simulation education provides repetitive learning opportunities within a safe environment that does not compromise patient safety. This approach fosters learners’ knowledge, skills, and attitudes while promoting satisfaction and confidence during the learning process. However, conventional CPR simulation training often requires expensive equipment, including manikins and defibrillators for installation and maintenance [12]. Also, the management of emergency carts in hospital wards entails designated personnel conducting regular checks on inventory, medication verification, and expiration dates. Consequently, access to these carts is challenging unless there is a specific purpose. Limitations in environmental depiction can diminish learner engagement, and constraints on time and space can restrict repeated practice [12]. Thus, within economic constraints, an efficient educational approach is needed to transcend temporal and spatial limitations and allow nurses to sustain the effectiveness of CPR training.

Mixed reality (MR) has recently gained attention due to innovative advancements in digital technology. MR integrates virtual information into the real world, allowing learners to experience immersive clinical environments with virtual elements incorporated within real educational spaces [13]. Moreover, multi-user accessible MR simulations have been identified as a solution to overcome the technical limitations of virtual or augmented reality, enabling team-based training without the constraints of time or space [14]. In the traditional CPR training, where instructors lead practical sessions and learners follow by practicing while watching videos, the predominant approach faces limitations when conducting team-based simulations. Given the diverse and complex procedures involved in CPR, team-based simulation education on non-technical skills such as team work and leadership is emphasized as it significantly impacts CPR outcomes [8]. Effective education in team-based CPR training is achieved when team members adhere to their respective roles and engage in interaction [15]. Building upon the commonly used mannequin simulation, MR simulation, integrating mixed reality technology, allows for guidance on each role, facilitating smooth communication during CPR through features like sharing electrocardiogram monitors and emergency carts. Although there are technical limitations in fully implementing MR, recent research indicates the application of simplified team-based simulations through direct communication channels established between mentors and mentees using hololens [16]. This technology delivers information through visual, auditory, and tactile sensations, encouraging user participation in simulations. The integration of MR technology into team-based emergency nursing simulations has the potential to be a valuable tool for training and enhancing the emergency nursing response skills and decision-making demanded in real clinical settings.

Therefore, this study aims to develop and implement team-based emergency nursing simulations for clinical nurses using MR. We aim to ascertain the effects on critical thinking, clarity of communication, communication confidence, learning transfer motivation, and immersion in simulation during emergencies. The goal is to present a novel approach to nursing education by utilizing MR, enhancing nurses’ emergency patient response capabilities.

### Theoretical framework

This study designed emergency nursing simulations using MR based on NLN Simulation Theory [11] and principles for virtual reality-based educational simulations [17]. The simulation experience is shaped within the overall context, of the learning background and design. A key concept is the dynamic interaction between the facilitator and the participants, presenting outcomes of the simulation process [11].

Therefore, we aimed to establish appropriate learning objectives during the simulation design process by considering the learning background and design. To enhance engagement and fidelity in the learning process, we sought the participation of nursing faculty with expertise in emergency clinical practice, experienced nurses, and developers for technical utilization. Popup windows within the simulation and team collaboration problem-solving stages were introduced to enable learners to perform appropriate emergency nursing interventions. Subsequently, team-based and individual debriefing sessions were carried out.

To develop a simulation program incorporating the novel MR digital technology, this study followed the design principles of Han and Lim [17] in the completion of the prototype development phase. These design principles encompassed 12 stages to combine real-world and virtual information. The principles included alignment with real-world issues, technological suitability, similarity to real-world environments, structural planning, expert-driven implementation, activity progression, simplicity and complexity, virtual perception, realism in manipulation and selection, information provision, cognitive stimulation, and reflective contemplation. Following these guidelines, real-world and virtual information integration was conducted, and the prototype was iteratively evaluated. The design phases aimed to enhance dynamic interaction between facilitators and participants and increase the potential for use in education.

For the efficient operation of this program, instructors secured clinical expertise through more than 15 sessions of MR and emergency nursing simulation training experiences. The learners were nurses who encountered cardiac arrest patients within the hospital and who had undergone CPR training.

The team-based emergency nursing simulation education utilizing MR was designed to foster active learning, feedback, cooperative learning, interactive learning, and experiential learning environments to enhance interaction between facilitators and participants.

## Materials and methods

### Study design

A nonequivalent control group pretest-posttest design was utilized to compare the effects of a team-based MR simulation program on emergencies between an experimental and a control group.

### Participants and sample size

This study targeted 64 nurses in three hospitals in D city. To determine the required sample size, G*power 3.1.9.4 software was employed. The calculation was based on an independent t-test for the experimental and control groups.

Drawing upon a meta-analysis study on the effects of VR, AR, and MR-based learning in Korea [18], an overall effect size of 0.873, a power of 0.90, and a significance level of .05 were applied. As a result, the calculated sample sizes were 29 participants for each group. Considering potential attrition, 32 participants were selected for the experimental and control groups, yielding 64 participants. After considering the exclusion of one participant with missing questionnaire responses, one participant who withdrew from participation due to personal reasons, and one participant who did not complete the post-survey, a total of two participants from the experimental group and one from the control group were excluded. Consequently, the final analysis included 30 participants from the experimental group and 31 from the control group (S1 Fig).

The inclusion criteria for participants were nurses working at a tertiary general hospital who agreed to participate in the study. Exclusion criteria included nursing managers who were not directly involved in clinical nursing duties and newly hired nurses with less than six months of experience, as their limited experience could potentially impact the study results.

### Data collection

The study was conducted after obtaining written-approval from the Bioethics Review Committee of Kyungpook National University (KNU-2023-0072). The study period was from June 12 to August 11, 2023. The recruitment targeted nurses employed at a comprehensive hospital located in D city. After disseminating recruitment notifications, potential participants who expressed their willingness to participate in the study were provided with a comprehensive explanation of the research objectives. Written consent was then procured from those who voluntarily agreed to participate. The informed consent delineated pertinent details about the researchers, the study’s objectives, methodologies, and other relevant information. It assured participants that their data would be encrypted and exclusively employed for research purposes. Moreover, the implementation of distinct identification numbers ensured participant anonymity.

The experiment and control groups were separated based on program dates and durations to minimize contamination between groups. Upon completing the survey, participants received suitable incentives. They were informed that they were free to notify the researchers in case they encountered eye fatigue during the program, and they could request breaks or discontinue their involvement at any point with no repercussions.

### Simulation in MR program in emergency situations

Utilizing MR, team-based emergency situation simulation has been developed using the software construction method known as Extreme Programming [19], which involves building software based on continuous feedback. The educational content for team-based emergency situation simulation refer to the Korean Advanced Life Support (KALS) guidelines [20] and cardiopulmonary resuscitation (CPR) guidelines [1, 8] for the development.

The program objectives, specific contents were designed based on the opinions of four experts, including three nursing professors, one emergency medicine professor, and one anesthesiology and pain medicine professor. The designed program content required two emergency room nurses, one nursing professor, and one nursing education specialist to score each domain. Team-based emergency situation simulation education comprised four sub-domains, including the emergency cart and equipment area, electrocardiogram area, procedure algorithms and performance guide area, and communication area, with a total of 13 domains. Each domain was evaluated on a scale from 1 to 4, representing ‘Very Inappropriate’, ‘Inappropriate’, ‘Appropriate’, and ‘Very Appropriate’. The content validation was developed based on the criteria proposed by Polit et al. [21], and the content validation results indicated a Content Validity Index (CVI) of .80 or higher for all questions.

**Fig 1:**
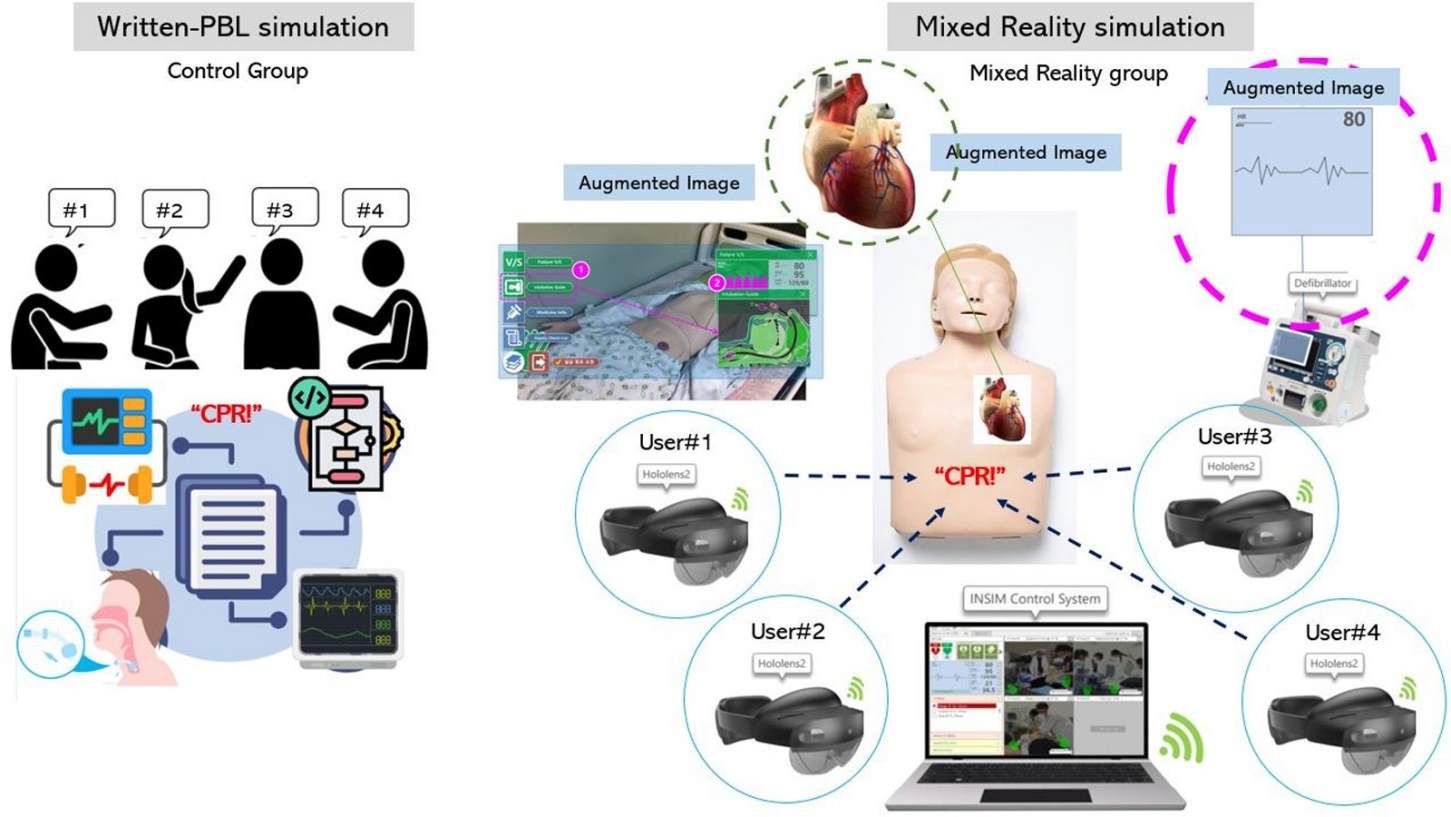
Design of the team-based MR simulation in emergency situation.

**Fig 2:**
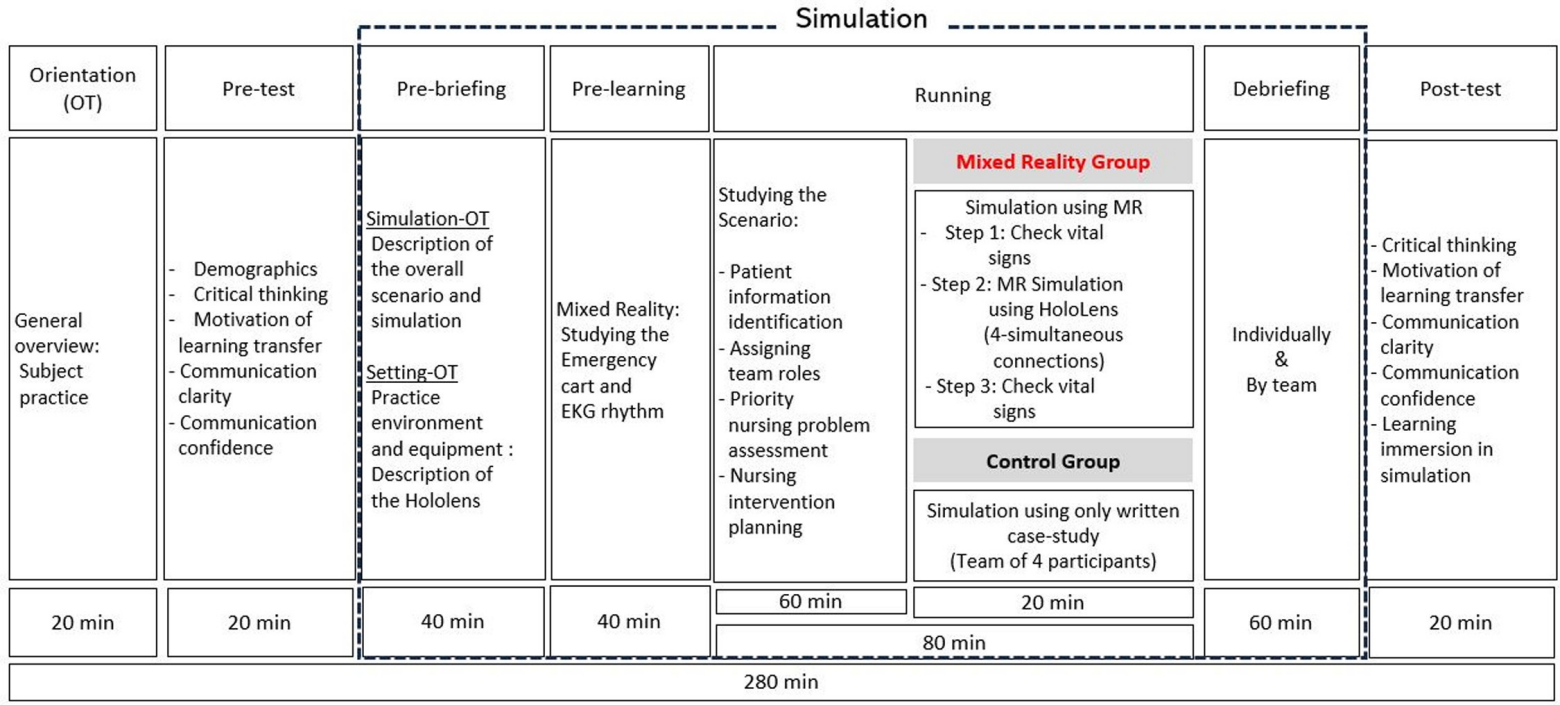
Study design.

The team-based emergency simulation program utilizing MR is designed to enable responses in CPR situations simultaneously using four HoloLens devices. Learners are assigned roles in advance and performed emergency simulation practices in groups of 4, each adhering to their designated roles. In the experimental group, participants were randomly assigned to groups of four individuals each, and the MR simulation program was conducted in the following steps for each team. In the first step, an orientation (OT) phase was implemented to provide an overview of the overall procedure. Subsequently, a pre-test was administered through a survey. The third step involved a pre-briefing phase, during which a 40-minute session was dedicated to orienting participants to the simulation and providing guidance on adapting to the Hololens. Through the setting-OT, participants were given the opportunity, before the actual operation of the MR simulation, to confirm Hololens guidelines and have free practice time to adapt to the Hololens. The fourth step consisted of a pre-learning phase. Which spanned a total of 40 minute. In this phase, augmented reality images are displayed on the HoloLens for learners to study the contents within the emergency cart, including medications and other items. A visual representation of a heart shape on the manikin was created to allow visualization of changing heartbeat rhythms according to electrocardiogram (ECG) readings (Fig 1). In running phrase, the team-based scenario learning was conducted for 60 minutes, followed by a 20-minute operation of the MR simulation. By interacting with the patient, learners can comprehend the patient’s condition stablished by the instructor, assign roles within groups, and practice communication similarly to real scenarios. A content guide processor through the HoloLens aids sequential practice according to different scenarios. The manikin, emergency cart, and defibrillator were tracked to facilitate interaction with actual equipment, enhancing the immersion and individual capacity. The trainee wearing the HoloLens experiences a simulation that parallels a real-life experience. Additional guidance and supplementary data are provided through the HoloLens for extended learning. Furthermore, the shared content visible to all four trainees, allows the participants to engage in the simulation while freely communicating (Fig 2). The final stage involved a debriefing phase, lasting a total of 60 minutes, which included individual debriefing as well as team debriefing. The control group followed the same procedural stages as the experimental group. However, during the running phase, rather than utilizing the MR simulation program, the control group received written CPR simulation materials. These materials delineated guidelines for each role within the emergency team of four individuals, assigned specific roles to each participant, and facilitated a written problem-based learning (PBL) study for each role.

### Measurement

#### Critical thinking

Critical thinking refers to the inclinations and patterns employed by learners in problem-solving and decision-making processes [22]. A tool crafted by Yoon [22] was employed for this investigation. The measurement comprised 27 questions, each evaluated on a 5-point Likert scale. Higher scores are indicative of more robust critical thinking dispositions. In Yoon’s study [22], the Cronbach’s α coefficient was recorded as .84, and it was recorded as .80 in this study.

#### Motivation of learning transfer

Motivation of learning transfer refers to applying acquired knowledge from educational training programs to different work contexts. In this study, the tool translated by Jung [23] from Holton, Bates and Rouna’s Learning Transfer System Inventory (LTSI) model [24] was utilized to measure learning transfer motivation. The measurement comprises five items rated on a 5-point Likert scale, with higher scores indicating greater levels of learning transfer motivation. In the original study by Jung [23], Cronbach’s α was .85, and it was .75 in this study.

#### Communication clarity

Communication clarity was evaluated employing a 5-point Likert scale consisting of 14 items, based on a modified and enhanced version by Cho [25] derived from a tool established by Marshall, Harrison, and Flanagan [26], initially comprising 20 items. Enhanced scores on this scale are indicative of improved communication clarity. In the investigation conducted by Cho [25], Cronbach’s α was .77, and it was .71 in the present study.

#### Communication confidence

The measurement for assessing communication confidence involves a standardized SBAR instrument (Situation, Background, Assessment, Recommendation), which quantifies the confidence exhibited by healthcare professionals while reporting clinical scenarios based on the standardized SBAR content. Developed by Kim [27], the measurement comprises five items and is scored on a scale from 0 “Not at all confident” to 10 “Extremely confident”. Higher scores indicate greater confidence in communication. In Kim’s study [27], Cronbach’s ɑ was .95, and it was .86 in this study.

#### Learning immersion in simulation

Using simulation-based learning immersion improves learners’ inherent motivation and fosters dynamic engagement, consequently optimizing learning achievements while mirroring real-world contexts. This study employed learning immersion in a simulation tool developed and validated by Ko [28]. This measurement is structured around four key facets: seven items of cognitive assimilation, three items of presence, three items of attention, and three items of self-determined experience. The scores were on a 5-point Likert scale that denoted escalated degrees of simulation-based learning immersion. Cronbach’s α was .89 in Ko’s study [28] and it was .93 in this study.

### Data analysis

The collected data were analyzed using SPSS 25.0 program. Descriptive statistics were used to present the participants’ general characteristics, including percentages, means, and standard deviations. Normality testing was conducted using the Shapiro-Wilk method, and for variables that did not meet the criteria of normality, non-parametric tests were employed. Homogeneity testing for general characteristics and dependent variables was conducted using chi-square tests and Mann-Whitney U test. Intergroup comparisons were performed using Mann-Whitney test to examine the differences in program effects.

## Results

### Homogeneity test of general characteristics and variables between groups

The homogeneity of the general characteristics and variables of the study subjects is presented in Table 1. The experimental group comprised 6 males (20.0%) and 24 females (80.0%), while the control group included 7 males (22.6%) and 24 females (77.4%), showing no statistically significant difference between the two groups (*p* = .806). The average ages were 26.80 (±2.95) years in the experimental group and 26.48 (±2.45) years in the control group, with no statistically significant difference between the two groups (Z = -0.31, *p* = .758). There were no significant differences between the two groups in terms of education level (χ^2^ = 0.08, *p* = .785), current department work experience (χ^2^ = 4.25, *p* = .236), simulation experience (χ^2^ = 0.14, *p* = .705), or CPR self-confidence (Z = -1.52, *p* = .129), and CRP satisfaction (Z = -0.38, *p* = .708), indicating homogeneity. The pre-study variables in the experimental and control groups passed the homogeneity test (Table 1).

**Table 1:** General characteristics of the participants. (N = 61).

Characteristics	Categories	Exp. (n = 30)	Cont. (n = 31)	χ^2^ or Z	*p*
		n(%) or M±SD	n(%) or M±SD		
Gender	Male	6 (20.0)	7 (22.6)	0.06	.806
	Female	24 (80.0)	24 (77.4)		
Age (yrs)		26.80±2.95	26.48±2.45	-0.31	.758
Education level	Bachelor’s degree	25 (83.3)	25 (80.6)	0.08	.785
	Post-graduate level	5 (16.7)	6 (19.4)		
Current department	Internal medicine ward	4 (13.3)	11 (35.5)	4.25	.236
	Surgical ward	18 (60.0)	14 (45.1)		
	ICU	3 (10.0)	3 (9.7)		
	ER	5 (16.7)	3 (9.7)		
Nurse work experience (month)		41.4±24.30	37.35±17.78	-0.61	.544
Current department work experience (month)		31.17±13.18	33.90±16.64	0.21	.834
Simulation experience	Yes	17 (56.7)	16 (51.6)	0.16	.692
	No	13 (43.3)	15 (48.4)		
VR experience	Yes	15 (50.0)	17 (54.8)	0.14	.705
	No	15 (50.0)	14 (45.2)		
MR experience	Yes	0 (0.0)	0 (0.0)		
	No	30 (100.0)	31 (100.0)		
CPR self-confidence		4.33±1.47	4.77±1.43	-1.52	.129
CPR satisfaction		5.83±1.70	5.61±1.63	-0.38	.708
Critical thinking		3.30±0.23	3.30±0.21	-0.23	.816
Motivation of learning transfer		3.85±0.45	3.78±0.36	-0.30	.766
Communication clarity		3.50±0.27	3.53±0.26	-0.68	.500
Communication confidence		6.34±0.62	6.32±0.61	-0.36	.721

Table notes Exp. = Experimental group; Cont. = Control group; VR = Virtual Reality; MR = Mixed Reality; CPR = Cardio-pulmonary resuscitation; ICU = Intensive Care Unit; ER = Emergency Room

### Effectiveness of team-based MR simulation program

The effects of the team-based emergency simulation program using MR are presented in Table 2.

**Table 2:** Effectiveness of team based MR simulation program. (N = 61).

Variables	Exp. (n = 30)	Cont. (n = 31)	Z	*p*
	M±SD	M±SD		
Critical thinking	3.97±0.35	3.45±0.40	-4.35	< .001
Motivation of learning transfer	4.24±0.31	3.92±0.52	-2.75	.006
Communication clarity	3.77±0.39	3.62±0.48	-1.43	.152
Communication confidence	7.99±0.50	7.40±1.23	-2.14	.033
Learning immersion in simulation	4.01±0.40	3.52±0.45	-4.40	< .001

Table notes MR = Mixed Reality; Exp. = Experimental group; Cont. = Control group

The critical thinking scores for the experimental group were 3.30 (±0.23) points before the program and 4.01 (±0.40) points after the program, while the control group had scores of 3.30 (±0.21) points before and 3.52 (±0.45) points after. The differences between the two groups were statistically significant (Z = -4.35, *p* < .001).

The motivation of learning transfer scores showed statistically significant differences, with the experimental group having scores of 3.85 (±0.45) points before and 4.24 (±0.31) points after the program, while the control group had scores of 3.78 (±0.36) points before and 3.92 (±0.52) points after (Z = -2.75, *p* = .006). Communication clarity scores showed no statistically significant difference between the experimental 3.77 (±0.39) and the control group 3.62 (±0.48) (Z = -1.43, *p* = .152). Communication confidence scores exhibited statistically significant differences, with the experimental group scoring 6.34 (±0.62) points before and 7.99 (±0.50) points after the program, while the control group scored 6.32 (±0.61) points before and 7.40 (±1.23) points after (Z = -2.14, *p* = .033). The learning immersion in simulation scores of the experimental group participants were 4.01 (±0.40) points, significantly higher than those of the control group with 3.52 (±0.45) points (Z = -4.40, *p* < .001).

## Discussion

This study aimed to develop a team-based emergency simulation using MR and validate its effectiveness among nurses. The study aimed to demonstrate the usefulness of a simulation 0educational approach integrating new digital technologies.

The experimental group that underwent team-based emergency simulation exhibited significantly higher levels of critical thinking than the control group. These findings align with prior research results [29] indicating enhanced critical thinking among nursing college students following mixed reality education. Critical thinking is necessary for learners to logically analyze clinical situations and execute appropriate nursing actions based on scientific evidence [22, 30]. According to Chen et al. [31], VR simulation’s limitations stem from providing information solely within all virtual environments, leading to issues with information direction perception and a high cognitive load for learners, potentially hindering the enhancement of the critical thinking process. However, MR simulations prove efficient in recognizing the physical cues of patients and attempting clinical decision-making [16]. The MR simulation developed in this study is believed to have enhanced learners’ critical thinking for problem-solving by amalgamating virtual and real-world information, thereby reducing the cognitive load. In addition, critical thinking is recognized as a cognitive tool [32] essential for problem-solving and decision-making in the context of MR simulations, allowing the acquisition of knowledge when necessary. This, in turn, contributes to heightened confidence in decision-making during practical actions. The utilization of technologies such as VR and MR to enhance cognitive functions is believed to be particularly effective, given their capacity to present stimulus information in a clear three-dimensional format [33, 34]. Furthermore, the post-simulation debriefing process provided an opportunity to reassess the accuracy of judgments made in response to situations encountered during the MR simulation. This reflective process is perceived as a means to enhance nurses’ autonomy and problem-solving skills, ultimately leading to the improvement of critical thinking abilities.

The results of this study indicated that the experimental group showed a significantly higher motivation level of learning transfer than the control group. According to previous research, team-based simulation education increases observational and experiential learning opportunities, leading to learning transfer. It was found that by solving similar problems, learners’ perception of applying their knowledge in real situations is enhanced [28, 35, 36]. In this study, utilizing MR technology in team-based simulation education created an immersive environment that increased opportunities for relearning 4 times according to each role, thereby enhancing nurse’ learning transfer motivation to apply learned knowledge and skills to actual clinical practice. Moreover, to heighten the level of learning transfer, the impact of how learning content is utilized and applied is crucial. It has been suggested that the closer the stimulus aligns with real job situations, the more it can facilitate learning transfer [37]. Therefore, the implementation of MR simulations that replicate real-world scenarios played a significant role in achieving positive outcomes. Consequently, there is a need for simulation studies that incorporate diverse technological strategies to enhance the clinical realism of simulations, aiming to improve the applicability of education for nurses. Therefore, simulation studies incorporating various technological strategies to heighten the clinical realism of simulations are needed to enhance the applicability of education for nurses.

The results of this study revealed that the experimental group exhibited a significantly higher level of communication confidence than the control group. Effective communication among team members, such as circulatory communication in advanced cardiac life support and clear information delivery (relevant to CPR tools), is crucial. Prior research conducted with nursing students through team-based simulation education has shown that learners’ communication skills and confidence in communication improve [38, 39]. Previous MR studies reported that simulations designed for pediatric transport teams enhanced participants’ communication skills through experiential interaction within mixed reality environments [38]. Therefore, utilizing MR in team-based emergency simulations is believed to enhance learners’ communication confidence by offering interactive learning opportunities for discussion among learners, recalling colleague’s instructions, and practicing clear communication among team members while enacting scenarios.

The experimental group that participated in the team-based emergency simulation showed a significantly higher level of learning immersion in simulation compared to the control group. Education utilizing VR, AR, and MR has been found to enhance learners’ immersion and promote engagement [40]. In studies focusing on the experience of Holopatient, MR-based education was found to facilitate learners’ understanding by representing the patient’s condition in 3D [29]. The program used in this study provided learners with visual elements of emergency patients’ physiological status and medical equipment, tactile sensations such as chest compression depth and location, auditory cues such as patients’ breathing and heart sounds, and dynamic interactions among healthcare professionals. This comprehensive approach is believed to have elevated the learning immersion level through simulation. MR, by amalgamating virtual reality and augmented reality, allows the integration of virtual and real-world elements, enabling interactive experiences. From a learning perspective, MR simulations are believed to enhance immersion and realism, potentially increasing the retention effect. Consequently, it is thought that learning immersion scores are higher in MR simulations compared to written PBL study, due to the ability of MR to merge virtual worlds with reality [41].

Therefore, the team-based emergency simulation using MR in this study has demonstrated its utility as an educational program by enhancing nurses’ critical thinking, learning transfer motivation, communication confidence, and learning immersion in simulation. This underscores the need to expand simulation education using MR as an effective educational strategy to enhance the efficiency of emergency nursing education.

## Conclusion

This study develops a team-based emergency simulation using MR to replicate real-world hospital emergency scenarios, making it academically important. The program’s results also have practical relevance, notably strengthened clinical nurses’ preparedness for emergencies through enhanced critical thinking, learning transfer motivation, communication confidence, and learning immersion through simulation. Future research will aim to continuously perform technical and nontechnical verification studies on the effectiveness of the program in emergencies, contributing to the development of more systematic and effective learning strategies. Additionally, by developing diverse simulation programs utilizing MR, this study aims to contribute to designing simulation education models that enhance practical skills in real-world settings.

This study has several limitations. First, there is insufficient precedent research to establish reliability and effectiveness due to technical constraints in applying MR-based simulation. Second, while this study validated the outcomes by comparing the experimental and control groups, further research is needed to assess the long-term effects and practical applicability of the intervention in emergency situations.

## Supporting information

S1 Fig Flow chart in this study. (JPG)

S1 File Team based mixed reality simulation program dataset. (DOCX)
