# Supplementary figures and images for: Effects of team-based mixed reality simulation program in emergency situations
Source: PLoS One. 2024 Feb 29;19(2):e0299832. doi: 10.1371/journal.pone.0299832 (PMC10903827; doi:10.1371/journal.pone.0299832)

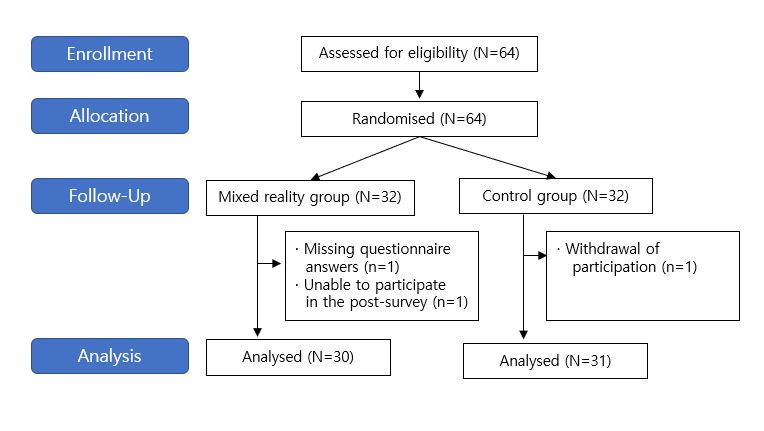

Supplement: S1 Fig — (JPG) [file pone.0299832.s001.JPG]
