# Supplementary material for: Effects of team-based mixed reality simulation program in emergency situations
Source: PLoS One. 2024 Feb 29;19(2):e0299832. doi: 10.1371/journal.pone.0299832 (PMC10903827; doi:10.1371/journal.pone.0299832)
Supplement: S1 File — (DOCX) [file pone.0299832.s002.docx]

**Supplementary information**

Table S1 General characteristics dataset of participants

| Groups | Gender | Age | Education level | Current department | Nurse work experience | Current department work experience | Simulation experience | VR experience | MR experience | CPR self-confidence | CPR satisfaction |
| --- | --- | --- | --- | --- | --- | --- | --- | --- | --- | --- | --- |
| 1  1  1  1  1  1  1  1  1  1  1  1  1  1  1  1  1  1  1  1  1  1  1  1  1  1  1  1  1  1  2  2  2  2  2  2  2  2  2  2  2  2  2  2  2  2  2  2  2  2  2  2  2  2  2  2  2  2  2  2  2 | 2  2  2  1  1  1  1  1  1  2  2  2  2  2  2  2  2  2  2  2  2  2  2  2  2  2  2  2  2  2  1  1  1  1  1  1  2  2  2  2  2  2  2  2  2  2  2  2  2  2  2  2  2  2  2  2  2  2  2  2  1 | 30  29  33  28  24  25  24  24  30  37  24  25  24  26  25  24  26  26  26  25  28  28  29  28  24  26  26  28  26  26  31  30  30  28  28  29  29  29  24  24  24  24  25  25  24  26  25  25  25  24  24  26  26  26  26  25  25  25  29  27  33 | 3  2  2  2  2  2  2  2  3  2  2  3  2  2  2  2  2  2  2  2  3  2  2  3  2  2  2  2  2  2  2  2  3  2  2  3  2  2  2  2  2  2  2  2  3  2  2  3  2  2  2  2  2  2  2  2  3  2  2  2  3 | 4  2  1  2  2  1  2  2  2  2  1  2  1  2  2  2  3  3  3  2  2  2  2  4  4  4  2  4  2  2  4  4  2  2  2  1  1  1  4  1  2  2  1  1  1  1  3  3  3  1  1  1  2  2  2  2  2  2  2  2  2 | 64  39  86  76  14  40  37  32  73  122  17  27  17  29  30  25  39  37  40  17  53  52  61  17  14  26  37  53  36  32  68  65  74  54  52  61  65  39  38  26  20  13  25  36  26  36  32  26  28  26  17  18  26  36  27  14  26  26  68  50  40 | 64  14  37  16  14  40  37  32  37  20  17  27  17  29  30  25  39  37  40  17  53  52  26  17  14  26  37  53  36  32  68  65  74  54  52  26  17  39  38  26  20  13  25  36  26  36  32  26  28  26  17  18  26  36  27  14  26  26  68  26  40 | 2  2  2  1  1  1  1  2  1  1  1  1  1  1  1  1  2  2  2  2  2  1  1  1  1  1  2  2  2  2  2  1  2  1  2  1  2  2  2  2  2  2  1  1  1  1  1  1  1  2  2  2  1  1  1  1  1  2  2  2  1 | 2  2  1  1  1  2  2  1  1  1  2  1  1  2  2  1  1  1  1  2  2  2  2  2  2  1  1  1  2  2  1  1  1  1  1  1  2  2  1  1  1  1  2  2  1  1  1  1  1  1  2  2  2  2  2  2  2  2  2  2  1 | 2  2  2  2  2  2  2  2  2  2  2  2  2  2  2  2  2  2  2  2  2  2  2  2  2  2  2  2  2  2  2  2  2  2  2  2  2  2  2  2  2  2  2  2  2  2  2  2  2  2  2  2  2  2  2  2  2  2  2  2  2 | 3  7  3  3  4  3  4  4  5  7  4  5  4  3  7  3  3  4  3  4  4  5  5  7  4  4  3  2  7  6  4  4  5  5  7  4  5  4  3  7  5  7  4  5  4  7  6  4  4  5  5  7  4  4  3  2  5  2  7  6  4 | 9  9  6  4  4  6  7  6  7  7  4  5  3  8  8  6  4  4  6  7  6  5  7  7  4  6  5  2  7  6  7  6  5  7  7  4  5  3  8  8  7  7  4  5  3  7  5  7  6  5  7  7  4  6  5  3  5  2  8  6  5 |

Table S2 Variables dataset of participants

| Group | Critical thinking | | Motivation of learning transfer | | Communication clarity | | Communication confidence | | Learning immersion in simulation |
| --- | --- | --- | --- | --- | --- | --- | --- | --- | --- |
|  | Pre-test | Post-test | Pre-test | Post-test | Pre-test | Post-test | Pre-test | Post-test | Post-test |
| 1  1  1  1  1  1  1  1  1  1  1  1  1  1  1  1  1  1  1  1  1  1  1  1  1  1  1  1  1  1  2  2  2  2  2  2  2  2  2  2  2  2  2  2  2  2  2  2  2  2  2  2  2  2  2  2  2  2  2  2  2 | 3.00  3.19  3.52  3.07  3.04  3.22  3.48  3.52  3.15  3.22  3.85  3.19  3.52  3.19  3.52  3.22  3.00  3.22  3.52  3.07  3.00  3.22  3.48  3.44  3.07  3.22  3.22  3.78  3.19  3.52  3.07  3.00  3.22  3.48  3.52  3.26  3.11  3.22  3.78  3.52  3.19  3.00  3.19  3.52  3.11  3.11  3.33  3.48  3.52  3.19  3.52  3.19  3.11  3.22  3.48  3.52  3.11  3.07  3.26  3.67  3.37 | 3.78  3.78  4.56  3.74  3.78  3.81  4.48  4.56  3.78  3.81  4.19  3.78  3.78  4.56  3.74  3.78  3.81  4.48  4.44  3.67  4.00  4.37  3.81  3.70  4.00  4.48  3.81  3.59  3.37  3.52  3.07  3.00  3.22  3.48  3.52  3.07  3.00  3.22  3.78  3.52  3.07  3.74  3.78  3.81  4.19  3.11  3.22  3.48  3.52  3.19  3.52  3.07  3.00  3.74  3.78  3.81  3.78  3.78  3.81  4.19  2.37 | 4.8  4  3.6  3.4  3.6  3.4  4  3.6  3.6  3.4  4  4.8  4  3.6  3.4  3.6  3.4  4  3.6  3.4  4  4.8  4  4  3.6  4.8  4  4  3.6  3.4  3.6  3.4  3.6  3.8  4  4.8  3.6  3.6  3.4  3.6  3.4  4  3.6  3.6  4  4.8  4  3.6  3.4  3.8  3.4  4  3.6  3.4  3.6  3.8  4  3.8  3.8  4  4.2 | 4.8  4  4  4.4  4.8  4  4  4  4.8  4  4.4  4  4.4  4.8  4  4  4  4.4  4  4  4.4  4.8  4  4.4  4  4  4.4  4  4  4.4  3.2  4  4.2  3.8  3.6  4  4.4  4  3.8  2.8  3.8  3.8  4  4.4  4  3.8  4.2  4.4  4.8  4  3.6  4  4.4  4  4  4  4.2  4  4  4.4  2 | 3.14  3.57  3.64  3.86  3.07  3.57  3.57  3.50  3.07  3.57  3.79  3.14  3.57  3.64  3.86  3.07  3.57  3.57  3.50  3.86  3.07  3.57  3.57  3.50  3.86  3.07  3.57  3.79  3.14  3.57  3.64  3.86  3.14  3.57  3.50  3.86  3.07  3.57  3.21  3.21  3.57  3.50  3.86  3.07  3.57  3.64  3.57  3.86  3.64  3.86  3.50  3.71  3.36  3.64  3.64  3.57  3.86  3.07  3.86  3.07  3.43 | 3.93  3.86  4.79  4.29  3.93  3.86  3.93  3.29  3.57  3.50  3.93  3.29  3.64  3.79  3.29  3.71  3.71  3.86  3.21  3.64  3.57  3.57  3.93  3.29  3.64  3.57  3.50  3.93  4.86  4.29  4.29  3.86  3.86  4.36  4.29  4.00  3.86  4.86  3.07  3.07  3.57  3.50  3.07  3.07  3.57  3.50  3.86  3.07  3.57  3.57  3.50  3.86  3.64  3.86  3.07  3.57  3.57  3.50  3.57  3.86  2.36 | 5.4  6.8  6.6  7  5.4  6.4  5.6  6.6  5.2  6.8  5.8  6.8  6.6  7.2  6.6  7  5.4  6.8  6.6  7  6  6.4  5.6  6.8  7  5.2  6.4  5.8  6.8  6.6  7  5.8  6.4  5.4  6.6  7  5.2  6.4  5.8  6.6  7  5.4  6.8  6.6  7  6.2  6.4  5.2  6.6  6.8  6.6  7  5.4  6.4  5.8  6.6  7  5.2  6.4  6.4  6.8 | 8.6  7.8  7  8.2  8.6  7.8  6.6  7  8.6  8  8.2  7.6  8  8.2  8.6  7.8  8  8  8.2  8.6  8.2  8.6  7.8  8  8  8  7.8  8.2  8.2  7.4  8.6  7.8  5.8  7  8  8.2  7.8  8  8.2  5.8  7.2  7  6.6  6.8  6.6  7  5.4  8.6  8  8.2  7.8  8  8.2  5.2  8.6  8  8.2  7.8  8  8  4.8 | 4.63  3.81  3.94  3.69  4.63  3.81  3.94  3.94  4.63  3.81  4.50  4.63  3.50  4.63  3.81  3.94  3.69  4.63  3.81  3.94  3.69  3.94  3.25  3.94  3.69  4.63  3.81  3.94  3.94  3.69  3.38  3.69  4.63  3.81  3.56  3.38  3.81  3.94  3.56  3.56  4.50  3.38  3.81  3.81  3.56  3.38  3.38  3.38  3.69  3.94  3.50  2.50  3.13  3.19  3.44  3.38  2.63  3.13  3.81  3.56  2.75 |
